# Enhancing sexual health in primary care: Guidance for practitioners

**DOI:** 10.4102/safp.v66i1.5822

**Published:** 2024-01-19

**Authors:** Padaruth Ramlachan, Keshena Naidoo

**Affiliations:** 1 International Society for Sexual Medicine (ISSM), Durban; and, African Society for Sexual Medicine (ASSM), Durban, South Africa; 2 Department of Family Medicine, University of KwaZulu-Natal, Durban, South Africa

**Keywords:** sexual health, primary healthcare, sexual dysfunctions, gender-diverse, screening

## Abstract

Sexual health is an integral aspect of overall health and well-being and is fundamental to the sustainable development of societies worldwide. The World Health Organization (WHO) defines sexual health as ‘a state of physical, emotional, mental, and social well-being in relation to sexuality’. However, addressing sexual health has been afforded low priority in primary healthcare systems. Primary care practitioners (PCPs), who play a crucial role in providing comprehensive care to communities, receive little training on screening and managing individuals with sexual health problems. The scope of services ranges from education, prevention and screening, to management of sexual health matters. Patients with noncommunicable diseases (NCDs), such as stroke, cancer, heart disease and diabetes, are at increased risk for sexual dysfunction, possibly because of common pathogenetic mechanisms, such as inflammation. This is of considerable importance in the sub-Saharan African context where there is a rapidly increasing prevalence of NCDs, as well as a high burden of HIV. Strategies to improve the quality of sexual health services in primary care include creating a safe and non-judgemental practice environment for history-taking among gender-diverse populations, utilising effective screening tools aligned with the Diagnostic and Statistical Manual of Mental Disorders (DSM-5) criteria for sexual dysfunctions. In particular, the International Consultation on Sexual Medicine (ICSM -5) diagnostic and treatment algorithm can empower primary care providers to effectively address sexual dysfunctions among patients and improve the quality of care provided to communities regarding sexual and reproductive health.

## Introduction

The concept of sexuality includes sex, gender identity, sexual orientation, intimacy, eroticism and pleasure as well as reproduction.^1^ Sexual health is an integral part of overall health and well-being and is fundamental to the sustainable development of societies worldwide. The World Health Organization (WHO) defines sexual health as ‘a state of physical, emotional, mental, and social well-being in relation to sexuality’.^1^ Sexual dysfunction, defined as ‘a significant disturbance in one’s ability to sexually perform or experience sexual pleasure’, is a common problem worldwide.^2^ In order to identify and address sexual health concerns, it is necessary to understand sexual function and common disorders.

## Understanding sexual function and dysfunction

The sexual response starts with sexual desire that is followed by arousal. Sexual arousal may lead a person to participate in sexually stimulating activities such as intercourse and masturbation. There are four phases of the sexual response cycle: desire (libido), arousal (excitement), orgasm and resolution.

Sexual dysfunctions are characterised by a significant disturbance in a person’s ability to respond sexually or to experience sexual pleasure. An individual may have multiple sexual dysfunctions concurrently, and all dysfunctions need to be addressed. The main classifications of sexual dysfunctions include female (sexual interest and arousal disorder, orgasmic disorder and genito-pelvic pain and penetration disorder) and male (desire disorder, erectile disorder, premature ejaculation and delayed ejaculation) sexual dysfunctions.^2,3^

Disturbances in desire, arousal, orgasm and pain that are present for 6 months or more and which cause distress require clinical care. Sexual disorders may be present lifelong or be acquired. They can also range in intensity from mild to severe and be situation-specific or generalised.

## Prevalence of sexual dysfunctions and risk factors

The Global Study of Sexual Attitudes and Behaviours, an international survey among adults aged 40–80 years across 29 countries, reported that sexual difficulties are relatively common between 40% and 45% of women and 20% and 30% of men throughout the world.^4^ Premature ejaculation and a difficulty in achieving and maintaining an erection were the problems reported most commonly by men, affecting 24% and 17%, respectively. The most commonly reported sexual dysfunction reported among women was a lack of sexual interest and an inability to reach orgasm, affecting 32% and 25%, respectively.^4^ Studies conducted in the South African context highlight the prevalence of sexual dysfunction among local communities. A study conducted in KwaZulu-Natal reported that the prevalence of erectile dysfunction (ED) prevalence was as high as 65% among adult males attending a primary care facility.^5^ Another study that was conducted among patients with chronic illnesses in South Africa reported that 98% of male clients had ED symptoms while up to 91% of female clinic attendees reported sexual dysfunction symptoms.^6^

People living with chronic non-communicable diseases (NCDs) such as diabetes mellitus and cardiovascular diseases are at significantly increased risk for sexual dysfunction.^7^ The estimated prevalence of ED in patients with diabetes in Africa was noted to be as high as 71.45%.^8^ Interestingly, ED has been identified as an independent marker for cardiovascular disease.^9^ In response to emerging evidence on the risk factors for sexual dysfunction, local guidelines have been adapted to include routine screening of people living with diabetes for sexual dysfunction.^10^ Other associated risk factors for sexual dysfunction include increased age, depression, mental health conditions, chronic pain, obesity, substance abuse, HIV and certain medications.^11,12^

Cancer is also commonly associated with adverse effects on sexual health, with up to 60% of women and up to 80% of men affected, regardless of cancer type.^13^ As life expectancy increases, the prevalence of comorbidities associated with sexual dysfunction will increase. Sexual dysfunction is known to adversely affect quality of life. In order to attain the Sustainable Development Goal target (Target 3.7) to ‘ensure universal access to sexual and reproductive healthcare services’, it is necessary that evidence-based guidelines on managing sexual health and dysfunction are implemented.^14^ It is therefore recommended that primary health providers address sexual health and functioning in all patients, especially those living with chronic diseases. However, there are significant challenges to providing sexual healthcare in primary healthcare systems despite the prevalence of sexual dysfunction.

## Challenges to sexual healthcare

The scope of sexual healthcare services ranges from education, prevention and screening, to management of sexual dysfunction. The delivery of sexual healthcare services at the primary care level should be person centred, respectful to all patients regardless of age, religion and ethnic origin, and sensitive to transgender and gender-diverse individuals. Primary care providers are ideally positioned to address the sexual health concerns of patients and enhance overall well-being.^14^ However, despite the availability of local guidelines on sexual history taking up to 75% of health professionals reportedly do not address their patients’ sexual health concerns.^15,16^ Factors for health professionals failing to identify and manage patients with sexual dysfunction could be time constraints, low awareness of sexual dysfunction, lack of training about diagnostic tools and treatment options, cultural barriers and fear of broaching the topic.^17,18^ In contrast, up to 91% of patients indicated that they would be comfortable if their health professional raised the issue.^18^ In addition, many people with sexual health concerns do not even attempt to seek medical help.^19^ Consequently, patients with sexual health concerns do not receive adequate care. It is thus imperative that health professionals not only include sexual health in routine history taking but help increase awareness of sexual health in communities. This is only possible if health providers are able and willing to address sexual health concerns in their consultations.

Unfortunately, there has been minimal inclusion of sexual health in most undergraduate medical and nursing curricula.^20^ Hence, most health professionals are inadequately prepared to address sexual health concerns in patients. Additionally, there have been few opportunities for postgraduate training in sexual health.

## Enhancing sexual healthcare

### Primary care providers

The knowledge gap on sexual healthcare needs to be addressed to ensure that primary care practitioners (PCPs) are better equipped to address sexual health concerns in their practice. In the context of high HIV-prevalence settings such as Africa, primary care providers should provide education on safe sex practices, contraception, sexually transmitted infections (STI) prevention and the options of pre-exposure prophylaxis (PrEP) and post-exposure prophylaxis (PEP) for HIV prevention. Health professionals have a key role in teaching patients about sexual healthcare and addressing barriers to accessing sexual healthcare such as stigma, discrimination, and lack of knowledge. Giving patients the ‘Sexual health First aid kit’ provides them with the competence to deal with ‘sexual accidents’, STI or pregnancy risks, when confronted with sexual dysfunction and when sexually assaulted.

### Creating a safe environment

Fostering a safe environment for patients to discuss sexual health is of utmost importance. Primary care practitioners should initiate the conversation and allow patients to decide whether they want to address their concerns and should be mindful of the diverse attitudes and practices related to sexuality in multicultural settings.

### Gender-sensitive considerations

Transgender and gender diverse individuals seeking primary healthcare should be respected and treated without prejudice. The primary care system in South Africa currently lacks resources and targeted programmes to cater for transgender people.^21^ This is, in part, because of the limited data on transgender and gender diverse populations which makes it difficult to develop guidelines and programmes. However, health professionals are tasked with ensuring that every patient is affirmed, respected and not judged. Recognising transgender and gender diverse people and understanding and accepting the large variety of sexual expression and identification is necessary for appropriate evaluation and management. Non-judgemental and direct questions best achieve this goal.

## Addressing sexual health in consultations

### Screening

There are a variety of validated screening tools for sexual dysfunctions that can be adapted to local contexts.^15,16,22,23^ Screening tools should identify a patient’s physical and emotional concerns regarding sexual health and should explore all aspects of sexual health. It is also essential that screening tools include patients of all sexual orientations. Self-administered screening tools, such as the International Index of Erectile Function (IIEF) and the Female Sexual Function Index (FSFI), are also available for patients to identify sexual dysfunction in men and women, respectively.^22,23^

### Five-step approach

**FIGURE 1:**
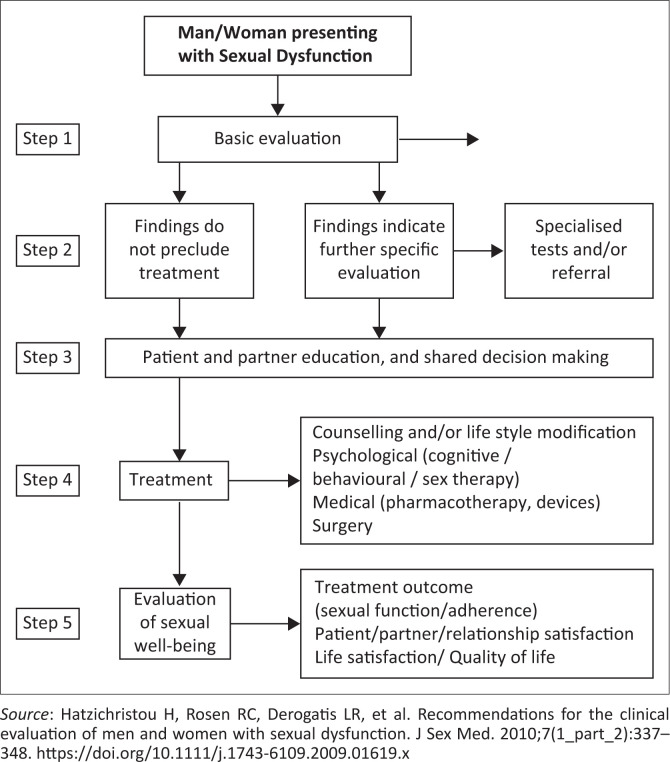
The International Consultation of Sexual Medicine–-5 (ICSM-5) Diagnostic and Treatment Algorithm (for men and women).

The International Consultation of Sexual Medicine-5 (ICSM-5) Diagnostic and Treatment algorithm provides a comprehensive and patient-centred approach to sexual health once sexual dysfunction is diagnosed (Figure 1).^16^ It consists of five steps: engaging, assessing, evaluating, planning and implementing and evaluating outcomes. Adapting the language and format of the tools to be inclusive and sensitive to diverse populations is crucial for accurate assessment.

Below is a summary of the five steps in addressing sexual health in a consultation.

#### Step 1: Engaging

Establishing trust, creating a safe and non-judgemental gender-sensitive environment and encouraging open communication with patients about their sexual health concerns.

#### Step 2: Assessing

Conducting a comprehensive assessment of sexual health, including medical, psychological, relational and sociocultural factors that may influence an individual’s well-being. Sexual, medical and psychosocial history is mandatory in every case.

#### Step 3: Evaluating

Conducting a physical examination and appropriate investigations are strongly recommended but not always necessary. Relevant information from the history can be used to identify potential sexual health problems, determine their impact on the individual’s overall well-being and assess the need for further investigation or intervention. There are limited specialised diagnostic procedures for women and are less widely used than those for men. Diagnostic procedures should only be used where there is compelling evidence to do so.

#### Step 4: Planning and implementing

Collaborating with the patient to develop a personalised care plan that addresses the identified sexual health concerns. This may involve medical interventions, psychoeducation, couple or single counselling, behavioural strategies or referrals to specialists as needed.

#### Step 5: Evaluating outcomes

Monitoring the progress and effectiveness of the interventions, reassessing the patient’s sexual health status and adjusting the care plan accordingly to optimise outcomes.

The International Consultation on Sexual Medicine (ICSM) algorithm serves only as a guide and framework, and the implementation should be tailored to the specific context, cultural considerations and resources available in each healthcare setting.

### Taking a comprehensive sexual history

A comprehensive sexual history-taking is vital to understand the underlying causes of sexual dysfunctions and propose appropriate treatment plans. Primary care practitioners should ask open-ended questions directed to satisfy diagnostic criteria of the Diagnostic and Statistical Manual of Mental Disorders (DSM-5), avoid assumptions and create a comfortable and confidential environment for patients to discuss their concerns. During the initial phase of assessment, physicians need to discriminate between sexual concerns, difficulties, dysfunctions and disorders. Cultural competency and sensitivity play a significant role, considering the diverse attitudes and practices related to sexuality in multicultural settings. Utilisation of national resources and guidelines aids is useful in providing culturally competent sexual healthcare.

Sexual history-taking should encompass sexuality risk assessment (5 P’s), sexual well-being status, medical conditions and their impact on sexual health, and care for special populations (LGBTQIa+) individuals, special needs and disability.^16,21^

### Assessment

1. Sexuality-related health risks (5 P’s) – Partners, Practices, Pleasure, Pregnancy plans, Past history/protection from STI, HIV and sexual violence.
2. Sexual wellbeing and dysfunction – Assessment of biological, sexual, relationship, psychological, cultural and social contributors to dysfunctions.
3. Assessment of the impact of comorbid disease on sexual health.
4. Assessment of special groups – those who are different and special needs groups, pregnancy, infertility, disability, colostomy, etc.

### Treatment approaches and overcoming challenges

Sexual health and dysfunction are complex and treating sexual dysfunctions requires a multidisciplinary approach addressing physical, psychological and relational factors. Psychoeducation and counselling, medication interventions and behavioural strategies are common treatment approaches. There are specific assessment and treatment guidelines for ED in men.^23^ Clinical guidelines have also been developed for evaluating and managing men with priapism and testosterone deficiency, as well as for disorders of libido, Peyronie’s disease, orgasm and ejaculation.^24^ Guidelines are also available for female sexual dysfunction, hypoactive sexual desire disorder (HSDD), menopause symptoms and androgen therapy.^25^

The complex aetiology of sexual dysfunction and associated risk factors, such as diabetes mellitus, cardiovascular disease, psychological factors, endocrine conditions and endothelial dysfunction warrants a careful approach to the care of patients with sexual health concerns (summarised in Table 1).

**TABLE 1:** Assessment of patients with sexual dysfunction.

Step1 (for men and women)
Address risk factors and comorbidities
Counsel patient and partner if possible
Initiate treatment based on – Patient Preference or Partners sexual function or Cardiovascular Risk (Men)

## Addressing sexual dysfunction

Management of patients with sexual dysfunction should be individualised to address the patient’s specific physical, psychological, interpersonal and socio-cultural concerns. Attention should be addressed to each area of sexual dysfunction, that is, desire, arousal, orgasm and pain. The available modalities for management range from education and psychotherapy, to lifestyle interventions, pelvic floor exercises, medications and mechanical devices.

Detailed management of sexual dysfunction in people living with diabetes will be available in the updated Society for Endocrinology, Metabolism and Diabetes of South Africa (SEMDSA) guidelines for the management of diabetes.^10^

### Male sexual dysfunction

Treatment of male sexual dysfunctions depends on the specific condition. For ED therapies include phosphodiesterase-5 inhibitors (e.g. sildenafil), vacuum devices, intracavernosal injections and penile implants.^24^ Newer therapies include topical prostaglandins, low-intensity shockwave therapy and platelet-rich plasma therapy.

Premature ejaculation may be treated with behavioural techniques and topical anaesthetics and off-label selective serotonin receptor inhibitors. Delayed ejaculation can be managed with psychotherapy or medication adjustments, whereas low libido might require hormone therapy and or counselling. Peyronie’s disease may necessitate surgery or collagenase injection.

### Female sexual dysfunction

The management of female sexual dysfunction necessitates a more comprehensive approach. Greater attention is required on education or counselling targeted at addressing performance anxiety and improving self-esteem (particularly in survivors of female genital mutilation or abuse). Some of the treatment modalities include cognitive behavioural therapy, systemic therapy, sensate focus and mindfulness. Patient education regarding self-discovery should cover guiding, sensate focus, fantasy, vibrator, erotic stimulation, lubricants and moisturisers

Other management aspects including risk factor modification, psychological therapy, hormonal adjustments (e.g. hormone replacement therapy) and self-discovery techniques. Strategies for risk factor modification include smoking cessation councelling, dietary advise, exercise therapy and weight management. Medication may be required to treat chronic pain, depression, pain, infections, vaginismus and partner dysfunction, while dose adjustments may be required for hormonal contraceptives, hormone replacement therapy in genito urinary syndrome of menopause, vaginal oestrogen and androgens (if indicated). A recent clinical practice guideline provides recommendations on the use of testosterone for women with HSDD.^25^

## Conclusion

Primary care practitioners play a crucial role in addressing the sexual health concerns of patients. By initiating conversations around sexual health, creating a safe and non-judgemental environment for history-taking, utilising evidence-based screening and management tools such as the ICSM-5 algorithm, PCPs can provide quality patient-centred sexual healthcare. Screening for sexual dysfunction in patients with chronic illnesses can promote a predictive personalised preventative participatory approach and lead to risk factor reduction. However, further research is needed in South Africa to explore the impact of sexual health recommendations on local communities. The WHO and professional associations must advocate for countries to include sexual health services as part of primary care. Multicomponent implementation programmes are needed to improve health professionals’ knowledge, competence and comfort when addressing sexual health.
